# Uncontrolled Diabetes as the Sole Risk Factor in a Rare Candida glabrata Tubo-Ovarian Abscess

**DOI:** 10.7759/cureus.105065

**Published:** 2026-03-11

**Authors:** Neha Iska, Victoria Pintar, Meryem Soylu, Ethan Dunn, Shagun Tuli, Jay Holmes, Atinuke L Akinpeloye, Panagiotis Cherouveim

**Affiliations:** 1 Obstetrics and Gynecology, Wayne State University School of Medicine, Detroit, USA; 2 Obstetrics and Gynecology, Hurley Medical Center, Michigan State University, Flint, USA

**Keywords:** candida glabrata, diagnostic delay, fungal tubo-ovarian abscess, pelvic inflammatory disease, uncontrolled type 2 diabetes mellitus

## Abstract

Fungal tubo-ovarian abscesses (TOAs) are rare and often mimic bacterial TOAs clinically and radiographically, frequently resulting in delayed diagnosis. Uncontrolled type 2 diabetes mellitus (T2DM) significantly increases susceptibility to fungal infection through impaired neutrophil function, tissue ischemia, and enhanced *Candida* colonization. Early recognition is critical, as standard empiric TOA regimens do not provide antifungal coverage. We describe the case of a 36-year-old patient with poorly controlled T2DM (hemoglobin A1c 15.6%) who presented with fever and right-sided abdominal pain. Imaging demonstrated a multiloculated right adnexal fluid collection concerning for TOA. Despite guideline-concordant broad-spectrum antibiotics and interventional radiology-guided drainage, the patient remained persistently febrile with minimal clinical improvement. Abscess aspirate cultures subsequently isolated *Candida glabrata*, prompting escalation of therapy from fluconazole to micafungin. Given the persistent collection despite prolonged antimicrobial and antifungal therapy, diagnostic laparoscopy with surgical drainage was performed, resulting in clinical resolution. This case highlights the importance of maintaining a high index of suspicion for fungal TOA in patients with refractory disease, particularly in the setting of uncontrolled diabetes, and underscores the need for early culture acquisition, timely initiation of anti-fungal therapy, and substantial consideration concerning operative source control.

## Introduction

In the United States, data support an increase in the incidence of pelvic inflammatory disease (PID) in recent years [1]. One of the most serious complications of PID includes the subsequent development of a tubo-ovarian abscess (TOA) in approximately 15-35% of patients hospitalized with PID [2]. TOA typically results from an ascending infection of the upper genital tract. Known risk factors for TOA formation include a past history of PID, history of sexually transmitted infections (STIs), multiple sexual partners, uterine instrumentation, and an early age of sexual debut, among others [3]. TOA can lead to acute life-threatening sepsis and hemorrhage. Furthermore, even treated TOA is associated with several potential long-term sequelae, including chronic pelvic pain, adhesions, and infertility [3-5].

TOA infections are typically polymicrobial and often precipitated by anaerobic or gram-negative bacteria colonization. The microbes most commonly implicated in TOA pathogenesis include *Neisseria gonorrhoea*, *Chlamydia trachomatis*, *Bacteroides*, *Peptococcus*, *Peptostreptococcus*, and *Escherichia coli* [6]. In rare circumstances, TOA can also be precipitated by fungal colonization; however, such cases have only been described in a small number of case reports, with causative organisms including *Candida albicans*, *Candida kefyr*, and *Candida glabrata *[7-11].

Fungal TOAs, like bacterial TOAs, can arise from multiple risk factors [9]. A common risk factor is diabetes mellitus, affecting 11% of the United States’ population and contributing to infection risk through immunosuppression [12]. In patients with uncontrolled diabetes mellitus, the immune system undergoes several changes, leading to decreased immunity, disruptions to the vaginal microbiome, and tissue ischemia [13-15]. Even though fungal TOAs are exceedingly uncommon, there have been cases associated with a variety of factors such as intrauterine device (IUD) use, prior gynecologic surgery, or chemotherapy-induced neutropenia; although, no cases have been reported in which uncontrolled diabetes mellitus was the sole precipitating factor [7-10,16,17].

Fungal TOAs can be particularly difficult to diagnose, as they present similarly to bacterial abscesses both clinically and on imaging. The empiric treatments for bacterial TOA do not cover fungal sources [18]. In refractory cases of TOA not responsive to empiric antibiotics within the first 48-72 hours, a fungal source should be suspected and surgical drainage for source control be considered, especially if initial vaginal cultures returned positive for yeast. Final diagnosis of a fungal TOA and the determination of a causative organism are made with cultures collected during abscess drainage [7,8].

We report what is, to our knowledge, the first documented case of TOA caused by *C. glabrata* in a 36-year-old female with type 2 diabetes mellitus as the only clearly identified risk factor, while acknowledging that unrecognized contributing factors may also have played a role. The present case highlights a critical need for increased clinical suspicion for fungal TOAs in patients with type 2 diabetes mellitus or any other systemic comorbidities that could potentially contribute to an immunocompromised state.

## Case presentation

We present the case of a 36-year-old gravida 3 para 2 African-American female who presented to the Emergency Department with three days of worsening right-sided intermittent abdominal pain, subjective fever, chills, and nausea. The patient denied any history of STIs or PID and was sexually active with one partner. She self-reported a history of frequent vaginal yeast infections, but no clinical diagnoses were available. The patient’s medical history was significant for poorly controlled type 2 diabetes mellitus, with a hemoglobin A1c (HbA1c) level of 15.6% on presentation. The patient denied taking her prescribed medication, which included metformin 1,000 mg twice a day and insulin glargine 35 units at bedtime. The patient’s surgical history was significant for two prior low transverse cesarean sections and concurrent bilateral tubal ligation with the Pomeroy technique three years prior to the encounter.

On presentation, the patient’s vital signs were as follows: body temperature 38.6°C, blood pressure 111/58 mmHg, pulse 108 beats per min, respiratory rate 16 breaths per min, SpO_2_ 95%, and BMI 44.9 kg/m^2^. Abdominal examination revealed tenderness in the right lower quadrant and right adnexal fullness, but there was no rebound tenderness or guarding. The genital examination was unremarkable, without cervical motion tenderness or vaginal discharge.

Initial laboratory results were significant for a white blood cell count of 15 cells/mm^3^ with 80% neutrophil, C-reactive protein 185.6 mg/L, and erythrocyte sedimentation rate 91 mm/hr (Table 1). Vaginal swabs were negative for *Chlamydia *and *Neisseria gonorrhoeae *and showed few yeast organisms on wet mount.

**Table 1:** Pertinent lab parameters

Lab parameter	Lab value	Reference value
White blood cell count	15.0 K/UL	4.0-10.8 K/UL
Neutrophil	80%	36-75%
C-reactive protein	185.6 mg/L	0-10 mg/L
Erythrocyte sedimentation rate	91 mm/hr	0-20 mm/hr
Hemoglobin A1C	15.60%	0-5.6%

**Figure 1:**
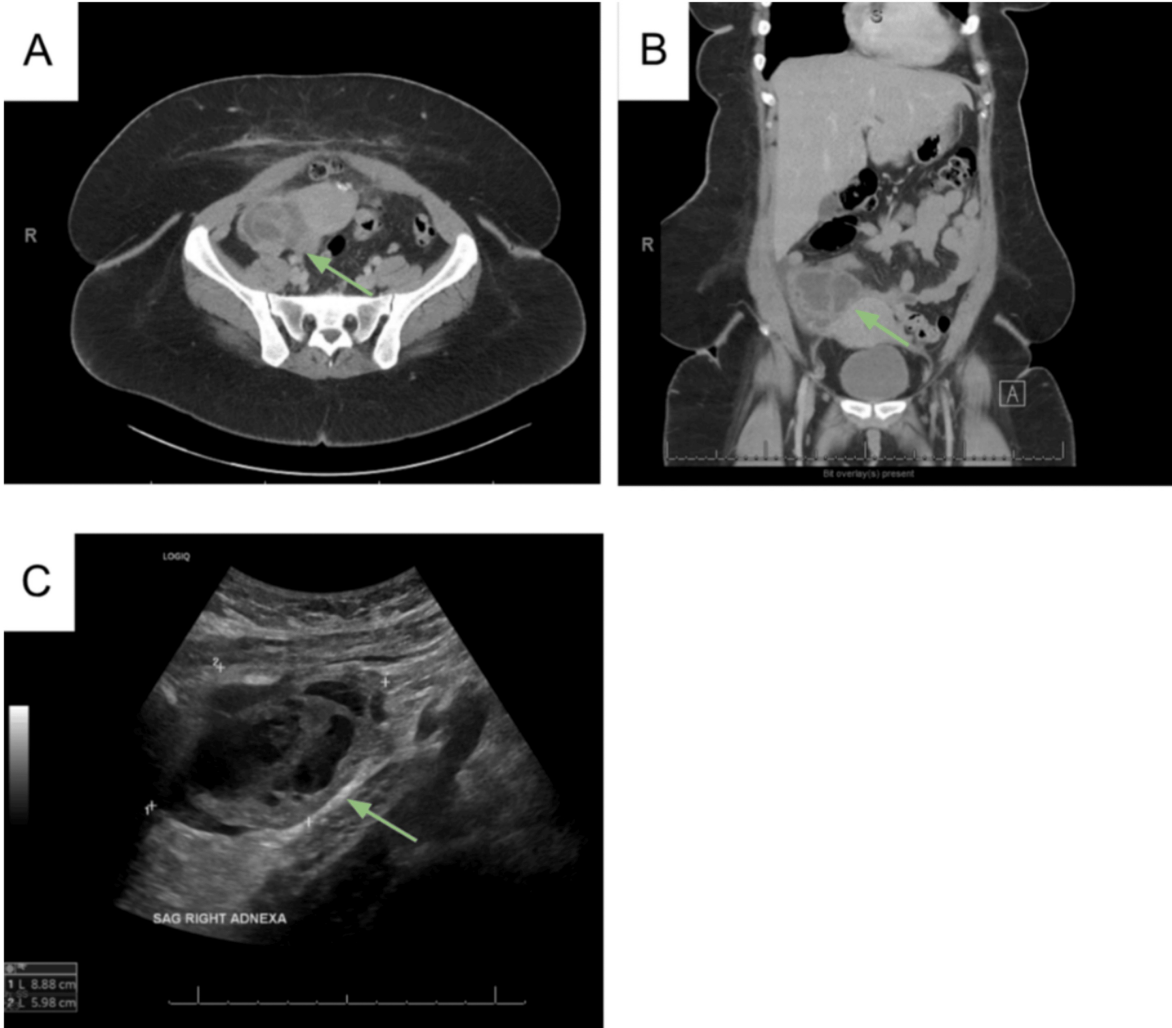
CT and ultrasound on initial presentation. CTAP with contrast showed a multiloculated right adnexal mass measuring 7.5 x 2.5 x 5.8 cm, concerning for a TOA and small calcified fibroid [axial (A) and coronal (B) planes]. Small right lower abdominal and pelvic free fluid. Pelvic ultrasound (C) showing a complex heterogeneous solid and cystic structure in the right adnexa measuring up to 8.9 x 6.0 x 8.1 cm with internal vascularity. CTAP, CT of the abdomen and pelvis; TOA, tubo-ovarian abscess

The CT of the abdomen and pelvis (CTAP) with contrast revealed a multiloculated right adnexal mass measuring 7.5 x 2.5 x 5.8 cm, concerning for a TOA (Figure 1). A small amount of pelvic free fluid was also identified. Pelvic ultrasound confirmed the presence of a complex heterogeneous solid and cystic structure in the right adnexa (8.9 x 6.0 x 8.1 cm) with internal vascularity.

The patient was subsequently admitted to our service and started on empiric intravenous (IV) antibiotic treatment with ceftriaxone 1,000 mg once daily, doxycycline 100 mg every 12 hours, and metronidazole 500 mg every 8 hours for suspected TOA. On hospital day 2, interventional radiology (IR) drainage was performed, the aspirated fluid was sent for cultures, and an accordion drain was placed. Despite drainage and treatment with empiric IV antibiotics, throughout days 1 to 4, the patient remained persistently febrile (Tmax 39.6°C) and in pain.

On hospital day 4, the fluid aspirate preliminary results were positive for yeast growth. Infectious disease was consulted, and fluconazole 400 mg once daily was added, while ceftriaxone was increased to 2,000 mg once daily. On day 5, cultures showed *C. glabrata*. Subsequently, fluconazole was switched to micafungin 100 mg injection once daily while waiting for sensitivities, given *C. glabrata’*s typical resistance to fluconazole. Doxycycline and metronidazole were switched to oral administration.

CTAP was repeated due to minimal drain output (5 mL/day) on hospital day 6 and revealed a persistent multiseptated fluid collection 7.6 x 5.1 x 5.9 cm, with anterolateral displacement of the pigtail drainage catheter. A fluoroscopically guided catheter exchange procedure was performed using a 12-French catheter on hospital day 9; drain output remained 0 mL/day. The patient remained afebrile, and repeat CTAP revealed an interval decrease in collection size to 5.3 x 4.5 cm. A peripherally inserted central catheter (PICC) line was placed, and the patient was discharged on hospital day 10 with the drain in place, with a plan for continued IV micafungin, oral amoxicillin/clavulanic acid, and doxycycline, as well as follow-up with infectious disease and repeat CT to assess for resolution.

**Figure 2:**
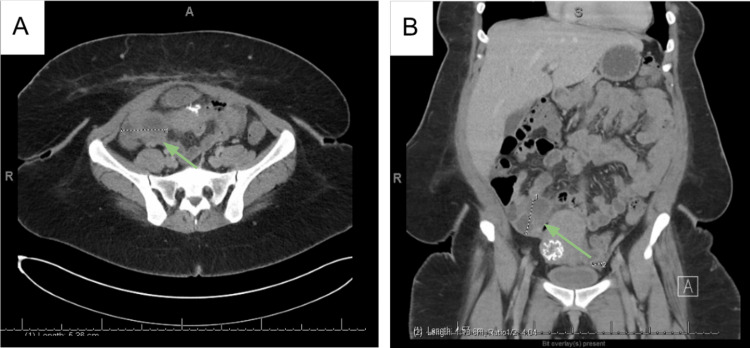
Follow-up CT scan 19 days after discharge from the hospital. CT of the abdomen and pelvis with contrast showed a multiloculated fluid collection measuring 5.3 x 4.5 cm in size [axial (A) and coronal (B) planes].

Repeat CTAP 19 days after hospital discharge showed a persistent multiloculated fluid collection (5.3 x 4.5 cm) (Figure 2). The drain was removed at that time by radiology as it was displaced with no output. Based on the CT results, the antibiotics and antifungal therapy were extended. On follow-up with infectious disease on post-discharge day 26, evaluation for surgical management with gynecology was recommended, given the minimal improvement with IR drainage and almost a month of medical therapy. At that time, doxycycline was discontinued, and IV micafungin and PO amoxicillin/clavulanic acid were continued in anticipation of follow-up with gynecology.

**Figure 3:**
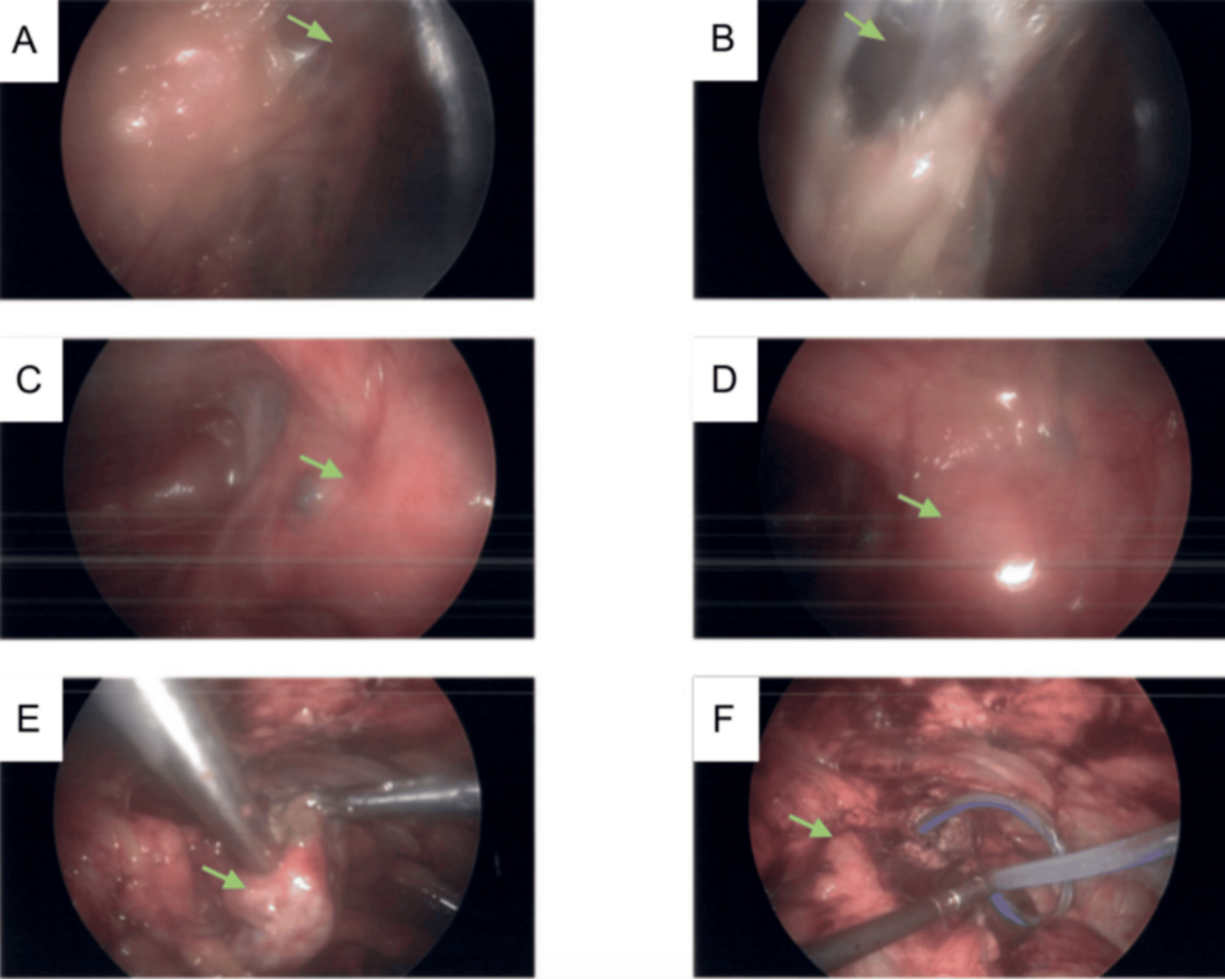
Intraoperative findings during laparoscopic TOA drainage. Adhesions were noted between the uterus and the anterior abdominal wall, the omentum, and the anterior abdominal wall (green arrow in A and B), as well as between the uterus and the anterior abdominal wall (green arrow in C and D), extending from the uterus to the falciform ligament and right TOA. Adhesions were lysed, and the right TOA was drained of thick green fluid and a Blake drain was placed (green arrow in E and F). TOA, tubo-ovarian abscess

After following up with gynecology, the patient was scheduled for diagnostic laparoscopy, which was carried out on post-discharge day 51. A full survey of the abdominal cavity revealed adhesions between the uterus and the anterior abdominal wall, the omentum, and the anterior abdominal wall extending from the uterus to the falciform ligament and right TOA (Figure 3). General surgery performed extensive lysis of adhesions, and the right TOA was drained of thick green fluid. Cultures were collected, and a Blake drain was placed into the cavity. The right fallopian tube and ovary were preserved. There were no complications to the procedure. Estimated blood loss was <5 mL.

The patient followed up in the gynecology clinic on post-operative day 4 after the Blake drain spontaneously came out, recovering well from the procedure. The drain output yielded serosanguinous fluid approximately 75 mL/day decreasing to 35 mL on post-operative day 4. Cultures collected during surgical drainage were negative. The patient was assessed by infectious disease demonstrating interval improvement without any acute symptoms or concerns; therefore, antibiotics were discontinued, and the PICC line was removed. At the four-week postoperative follow-up, the patient continued to feel well, reporting no complaints.

## Discussion

Fungal TOAs are significantly less common than bacterial and can present considerable diagnostic and therapeutic challenges. In the current case, the TOA culture revealed *C. glabrata* as the source of infection. Importantly, in recent years, the proportion of fungal infections caused by non-albicans *Candida *species has increased, particularly in critically ill or immunosuppressed patients [8,19].

Fungal TOAs may originate from ascending infections of the vagina or digestive tract [8]. This risk is exacerbated in patients with barrier disruption or systemic immunosuppression [8]. Fungal TOAs have been previously reported in association with IUDs, neutropenia, previous gynecologic surgery, and relative immunodeficiency [7-10,16,17]. In our patient, a significant predisposing factor could have been poorly controlled type 2 diabetes mellitus, with an HbA1c of 15.6%, indicating a chronic, persistent state of hyperglycemia. Both acute and chronic hyperglycemia impair immune system function through multiple mechanisms. Neutrophil function is diminished, reducing the immune system’s innate ability to phagocytose and clear *Candida *organisms [20]. Hyperglycemia also enhances the virulence of *Candida* pathogens due to glucosuria, providing nutrients for *Candida* organisms and subsequently facilitating colonization [8,21,22]. Furthermore, diabetes mellitus is associated with a higher prevalence of vulvovaginal candidiasis, with elevated HbA1C and glucosuria further increasing the risk [8,23-25]. Thus, our patient’s *C. glabrata* TOA could be attributed to extensive vaginal colonization, especially considering her self-reported history of recurrent yeast infections. It is possible that it may have initially been a mixed infection and that antibiotics eliminated bacterial organisms prior to initial cultures; however, with very little clinical improvement until the appropriate antifungal medication was started, it is most likely that *C. glabrata* was the main causative organism.

A major challenge in the management of fungal TOAs is diagnostic delay, largely due to their clinical rarity and similarity to bacterial TOAs. In our case, empiric antibiotic treatment proved ineffective, and the patient failed to improve until further diagnostic workup revealed *C. glabrata*. This result prompted a shift in the clinical approach and initiation of antifungal therapy.

Lack of clinical improvement within 48-72 hours of empiric antibiotic treatment should warrant further investigation and possibly surgical drainage. Surgical treatment is reserved for refractory cases of TOA and can entail laparoscopy, as in the present case, or possibly laparotomy, both of which are utilized to drain abscesses and lyse adhesions [26,27]. Because fungal TOAs are so rare, there is a lack of standardized approach to management. Further investigation into evidence-based guidelines for treatment duration, criteria for early surgical management, and treatment algorithms for fungal TOAs could be a valuable area of research in the future. In our case, earlier surgical management may have shortened the disease course and prevented long-term therapy with multiple antimicrobial medications.

This case is a rare presentation of a fungal TOA in a patient whose poorly controlled diabetes was the only clear risk factor and highlights the need for heightened clinical suspicion in TOAs refractory to empiric therapy. Clinicians should be encouraged to obtain abscess cultures in such patients to guide treatment and identify antifungal resistance. Given the limited research on fungal TOAs, timely culture acquisition and prompt initiation of antifungal therapy are critical to preventing delays in care and reducing long-term sequelae associated with this condition.

## Conclusions

In summary, we highlight some of the key features in the first reported case of *C. glabrata* TOA presenting in a patient with uncontrolled diabetes as the only clearly identifiable systemic immunocompromising factor. Although diabetes favors many infections, including vulvovaginal candidiasis, it is not commonly considered in the pathogenesis of fungal TOAs. This case highlights the importance of recognizing uncontrolled diabetes as a risk factor for fungal TOA development. Early consideration of fungal involvement in TOAs is critical for timely diagnosis and avoiding subsequent delays in care. Additionally, providers should be aware and counsel patients for the need for extended antimicrobial therapy and the possibility of eventually requiring surgery for source control. Here, we postulate that increased clinical awareness could reduce diagnostic delays, shorten hospital stays, and improve outcomes in patients affected by fungal TOAs. Since fungal TOAs are both rare and understudied, further research is required to better define diagnostic criteria, treatment algorithms, and precipitating factors.
